# Inhibition of macrophage migration inhibitory factor (MIF) suppresses apoptosis signal-regulating kinase 1 to protect against liver ischemia/reperfusion injury

**DOI:** 10.3389/fphar.2022.951906

**Published:** 2022-09-08

**Authors:** Sanyang Chen, Qiwen Yu, Yaodong Song, Zongchao Cui, Mengke Li, Chaopeng Mei, Huning Cui, Shengli Cao, Changju Zhu

**Affiliations:** ^1^ Department of Emergency Surgery, First Affiliated Hospital of Zhengzhou University, Zhengzhou, Henan, China; ^2^ Henan Medical Key Laboratory of Emergency and Trauma Research, Zhengzhou, China; ^3^ Henan Key Laboratory of Digestive Organ Transplantation, Zhengzhou, Henan, China; ^4^ Department of Hepatobiliary and Pancreatic Surgery, First Affiliated Hospital of Zhengzhou University, Zhengzhou, Henan, China

**Keywords:** MIF deficiency protects from hepatic I/R injury hepatic ischemia-reperfusion injury, macrophage migration inhibitory factor, apoptosis signal-regulating kinase 1, inflammation, apoptosis, oxidative stress

## Abstract

**Background:** Hepatic ischemia–reperfusion (I/R) injury is a major complication leading to surgical failures in liver resection, transplantation, and hemorrhagic shock. The role of cytokine macrophage migration inhibitory factor (MIF) in hepatic I/R injury is unclear.

**Methods:** We examined changes of MIF expression in mice after hepatic I/R surgery and hepatocytes challenged with hypoxia–reoxygenation (H/R) insult. Subsequently, MIF global knock-out mice and mice with adeno-associated-virus (AAV)-delivered MIF overexpression were subjected to hepatic I/R injury. Hepatic histology, the inflammatory response, apoptosis and oxidative stress were monitored to assess liver damage. The molecular mechanisms of MIF function were explored *in vivo* and *in vitro*.

**Results:** MIF was significantly upregulated in the serum whereas decreased in liver tissues of mice after hepatic I/R injury. MIF knock-out effectively attenuated I/R -induced liver inflammation, apoptosis and oxidative stress *in vivo* and *in vitro*, whereas MIF overexpression significantly aggravated liver injury. *Via* RNA-seq analysis, we found a significant decreased trend of MAPK pathway in MIF knock-out mice subjected hepatic I/R surgery. Using the apoptosis signal-regulating kinase 1 (ASK1) inhibitor NQDI-1 we determined that, mechanistically, the protective effect of MIF deficiency on hepatic I/R injury was dependent on the suppressing of the ASK1-JNK/P38 signaling pathway. Moreover, we found MIF inhibitor ISO-1 alleviate hepatic I/R injury in mice.

**Conclusion:** Our results confirm that MIF deficiency suppresses the ASK1-JNK/P38 pathway and protects the liver from I/R -induced injury. Our findings suggest MIF as a novel biomarker and therapeutic target for the diagnosis and treatment of hepatic I/R injury.

## Introduction

Hepatic ischemia–reperfusion (I/R) injury is associated with high morbidity and mortality in patients following liver trauma, liver resection or transplantation, and can cause hemorrhagic shock (Chen et al., 2021). Liver I/R injury accounts for up to 10% of early transplantation failures in liver transplantation, although surgical and allograft preservation techniques have been greatly improved (Kong et al., 2021). Unfortunately, despite its profound clinical importance, there are no effective treatments available for liver I/R injury, and clinical therapy is mainly limited to the prevention and control of risk factors (Guo et al., 2020; Guan et al., 2021). Therefore, thorough understanding of the underlying mechanisms and effective intervention measures to limit I/R-induced liver damage are urgently needed.

The mechanisms of hepatic I/R injury are highly complex and have been the focus of investigation for decades. Numerous previous studies show that inflammation, highly reactive oxygen species (ROS), nitrogen monoxide (NO) and cell death are the most critical factors associated with pathophysiology of hepatic I/R injury (Yuan et al., 2021; Zhang et al., 2021). During the ischemic period, the impeded blood supply results in an imbalance between metabolic supplements and demands, and causes eNOS failure. In the reperfusion phase, reoxygenation of the ischemic liver can stimulate the generation of excessive ROS through activated nicotinamide adenine dinucleotide phosphate oxidases. Meanwhile, the excess NO reacts with ROS to generate peroxynitrite (ONOO-), resulting in endothelial dysfunction and aggravating liver damage (Ferrer-Sueta and Radi, 2009; Zhang et al., 2022). Additionally, reperfusion causes the infiltration of neutrophils, macrophages and other inflammatory cells to the ischemic liver. The intense oxidative stress and inflammatory response can directly result in severe or even irreversible apoptosis, tissue injury, and organ dysfunction (Zhou et al., 2021a; Sahu et al., 2021). Therefore, determining strategies to suppress inflammation, oxidative stress and apoptosis could effectively ameliorate I/R-induced liver injury and contribute to the identification of novel pharmacological interventions to improve patient prognosis.

MIF was first discovered in 1966 as a pro-inflammatory cytokine that retains macrophages at sites of inflammation by preventing their random migration (Schindler et al., 2021). MIF is widely expressed in many cell types including lymphocytes, macrophages and non-immune cells such as hepatocytes, endothelial, epithelial, and tumor cells (de Azevedo et al., 2020). It is released from pre-formed storage pools when stimulated by inflammation. In addition to promoting inflammatory response, MIF displays various biological activities such as pro-apoptotic (Zhao et al., 2020), pro-oxidative-stress (Yang et al., 2020) and pro-angiogenic functions (Xu et al., 2008). Moreover, MIF has been reported to activate JNK and P38 in several disease, which play key roles during hepatic I/R injury. However, the function of MIF in hepatic I/R injury remains unknown.

The present study delineated that MIF plays an important role in hepatic I/R injury by aggravating inflammation, apoptosis, and oxidative stress *in vitro* and *in vivo*. Further molecular experiments showed that MIF deficiency could inhibit apoptosis signal-regulating kinase 1 (ASK1) activation and downstream JNK and p38 signaling during hepatic I/R injury. Our findings suggest MIF as a novel biomarker and therapeutic target for the diagnosis and treatment of hepatic I/R injury.

## Materials and methods

### Animals

Male C57 BL/6 mice and MIF knock out (KO) mice (C57 BL/6 background as previously described) (Zhu et al., 2020) aged 8–10 weeks (25 ± 2 g) were housed in a specific pathogen-free (SPF) facility under a controlled environment with 12-h light/dark photocycle (temperature, 23 ± 2°C). Food and water were available *ad libitum* throughout the study period. Animals received human care in adherence with the “Guide for the Care and Use of Laboratory Animals” prepared by the National Academy of Sciences and published by the National Institutes of Health (NIH; publication 86–23 revised in 1985). All animal procedures were approved by the Ethics Committee of The First Affiliated Hospital of Zhengzhou University. For studies in MIF overexpression in liver, we transduced adeno-associated virus (AAV) 8 system carrying GFP scramble (as a negative control) or MIF (designed and synthesized by Hanbio, Shanghai, China) into mice at a dose of 1 × 10^12^ vg (200 μl per mice) through tail-vein injection.

### Murine model of I/R injury

Partial (70%) liver warm ischemia mouse model was established as previously described (Chen et al., 2021). Briefly, mice were first anesthetized by pentobarbital sodium (60 mg/kg; Sigma) and subjected to midline laparotomy. A microvascular clip was used to clamp the left and middle portal vein and hepatic artery branches to interrupt the blood supply of the liver. After 1 h of ischemia, the clamp was removed for reperfusion. After 6 h reperfusion, the animals were sacrificed to collect liver and serum samples for further analysis. The residual blood was discharged by portal vein injection of normal saline. Part of the liver tissue was stored in liquid nitrogen and then transferred to the refrigerator at −80°C. Another part of liver tissue was preserved in 10% formalin for pathological examination. As a sham control group, mice underwent the same surgical procedure but without vasculature clamping. To inhibit MIF and ASK1 in mice, specific ASK1 inhibitor NQDI-1 (Sigma; 10 mg/kg, dissolution by DMSO and dilute with PBS to 1.25 mg/ml, 200 μl per mice) and MIF inhibitor ISO-1 (MedChemExpress LLC; 3.5 mg/kg, dissolution by DMSO and dilute with PBS to 0.44 mg/ml, 200 μl per mice) were intraperitoneally injected 2 h before the ischemic surgery. The same volume of DMSO (diluted to 0.1% by PBS) was used as control (Xu et al., 2020; Liu et al., 2021).

### Serum aminotransferase activities

Serum concentrations of alanine aminotransferase (ALT), aspartate aminotransferase (AST) and lactate dehydrogenase (LDH) were detected using the ADVIA 2400 Chemistry System (Siemens, Tarrytown, NY, United States) according to the manufacturer’s protocols.

### Liver hematoxylin and eosin and immunohistochemical staining

H&E staining was used to assess the necrosis of the liver. Paraffin-embedded sections of mouse liver tissues were sectioned to 4 μm slides. After deparaffinization and rehydration, the histomorphology analysis was carried out using H&E staining. For IHC staining, liver samples were dehydrated, paraffin embedded and sectioned to 4 μm slides. After incubating with primary antibodies of CD68 (GB11067, Servicebio, 1:50 dilution), Ly6g (GB11229, Servicebio, 1:50 dilution), and BAX (GB11007-1, Servicebio, 1:50 dilution) at 4°C overnight, the slides were washed and incubated with appropriate secondary antibodies conjugated with HRP were added for 1 h at room temperature. A 3,3′-diaminobenzidine (DAB) (ZLI-9032, Zhongshan Biotech, Beijing, China) was used to observed the sections followed by hematoxylin counterstaining. Images were visualized using a light microscope (Olympus, Tokyo, Japan).

### TdT-mediated dUTP nick-end labeling (TUNEL) staining

TUNEL staining was carried out to detect apoptosis in liver tissues according to the manufacturer’s protocol (Roche, 11684817910) as described previously (Pan et al., 2021).

### Enzyme-linked immunosorbent assay

The liver tissue sample was homogenized with homogenizer. ELISA was performed to detect the oxidative stress related factors malondialdehyde (MDA), superoxide dismutase (SOD), Glutathione (GSH) (ab118970, Abcam, Cambridge, United Kingdom) using commercial kits (MDA, ab118970, Abcam, Cambridge, United Kingdom; SOD, CSB-E08556m, CUSABIO, Wuhan, China; GSH, CSB-E13068m, CUSABIO, Wuhan, China) following the manufacturer’s instructions.

### RNA-seq analysis

Total RNA was isolated using mirVana miRNA Isolation Kit (Ambion) followingthe manufacturer’s protocol. RNA integrity was evaluated using the Agilent 2,100 Bioanalyzer (Agilent Technologies, Santa Clara, CA, United States). The libraries were constructed using TruSeq Stranded mRNA LTSample Prep Kit (Illumina, San Diego, CA, United States) according to the manufacturer’s instructions. Then these libraries were sequenced on the Illumina sequencing platform (HiSeqTM 2,500 or Illumina HiSeq X Ten) and 125/150 bp paired-end reads were generated. FPKM value of each gene was calculated using cufflinks, and the read counts of each genewere obtained by htseq-count. DEGs were identified using the DESeq (2012) R package functions estimateSizeFactors and nbinomTest.

### Cell lines culture and treatment

The mouse AML12 hepatic cell line was purchased from the Cell Bank of the Chinese Academy of Sciences. The cells were cultured in Dulbecco’s modified Eagle’s medium (DMEM) supplemented with 10% fetal bovine serum, and 1% penicillin-streptomycin. For the hypoxia-reoxygenation (H/R) model, cells were challenged with sugar-free, serum-free DMEM under hypoxia conditions (1% O2, 5% CO2, and 94% N2) in a modular incubator chamber (Biospherix, Lacona, NY, United States). After hypoxia for 6 h, the hepatocytes were returned to normal air conditions (95% air, 5% CO2) and medium incubating 6 h for reoxygenation. The full-length homo MIF cDNA was cloned into pHAGE-3×flag plasmids to express Flag-tagged MIF, Flag-tagged recombinant proteins. Several siRNA sequences for MIF were designed, and the knockdown sequences were constructed into the pLKO.1 vector. The primers used in this study are listed in Supplementary Table S3.

### Reactive oxygen species measurement

ROS production in mouse liver tissues were detected by DHE staining as previously described (Li et al., 2021). Dihydroethidium (DHE) can freely enter cells through living cells and be oxidized by ROS in cells to form oxyethidium; Ethidium oxide can be incorporated into chromosomal DNA to produce red fluorescence. In brief, fresh frozen liver sections (8 μm) were immediately incubated with 5 μM dihydroethidium (Invitrogen) at 37°C for 15 min. After the incubation, and then visualized by using a fluorescence microscope (Olympus DX51). Excitation and emission wavelength (DAPI ultraviolet excitation wavelength 330–380nm, emission wavelength 420nm, blue light; FITC excitation wavelength 465–495nm, emission wavelength 515–555 nm, green light; Cy3 excitation wavelength 510–560, emission wavelength 590 nm, red light).

### Quantitative real-time polymerase chain reaction

The mRNA expression in liver tissues and cell samples were detected using qRT-PCR as described previously (Chen et al., 2021). TRIzol reagent (Invitrogen) was used to extract total RNA. cDNA was reverse transcribed by using the HiScript^®^ III RT SuperMix for qPCR (+gDNA wiper) (Cat# R312, Vazme, Nanjing, China) and qRT-PCR was performed with ChamQTM SYBR qPCR Master Mix (Cat# Q311-02, Vazme, Nanjing, China). The mRNA levels were normalized against *ß*-actin expression. Primer sequences of the target genes are listed in Supplementary Table S1.

### Western blot

The protein levels in liver tissues and cell samples were detected using Western blot analysis as previously reported (Guo et al., 2020). Briefly, RIPA lysis buffer (65 mM Tris-HCl pH 7.5, 150 mM NaCl, 1 mM EDTA, 1% Nonidet P-40, 0.5% sodium deoxycholate and 0.1% SDS) with Protease and Phosphatase Inhibitor Cocktail (04693132001; Roche) was used to extract total proteins. Protein concentrations were detected using the BCA Protein Assay Kit (23225; Thermo Fisher Scientific) and were separated by SDS-PAGE, transferred to a nitrocellulose PVDF membrane. After incubation with primary and secondary antibodies, the blots were performed with enhanced chemiluminescence (ECL) reagents (170–5,061; Bio-Rad, Hercules, CA, United States) and captured by the ChemiDoc XRS + System (Bio-Rad). All the antibodies used in the western blot are listed in Supplementary Table S2.

### Statistical analysis

All data in the study was analyzed with SPSS (version 21.0, IBM, Armonk, NY, United States) and expressed as the mean ± SD. A two-tailed Student’s t test was used for comparisons between two groups and one-way analysis of variance (ANOVA) was used for comparisons between multiple groups. *p* < 0.05 was considered statistically significant.

## Results

### MIF expression is significantly increased in serum of mice subjected to hepatic I/R injury

**FIGURE 1:**
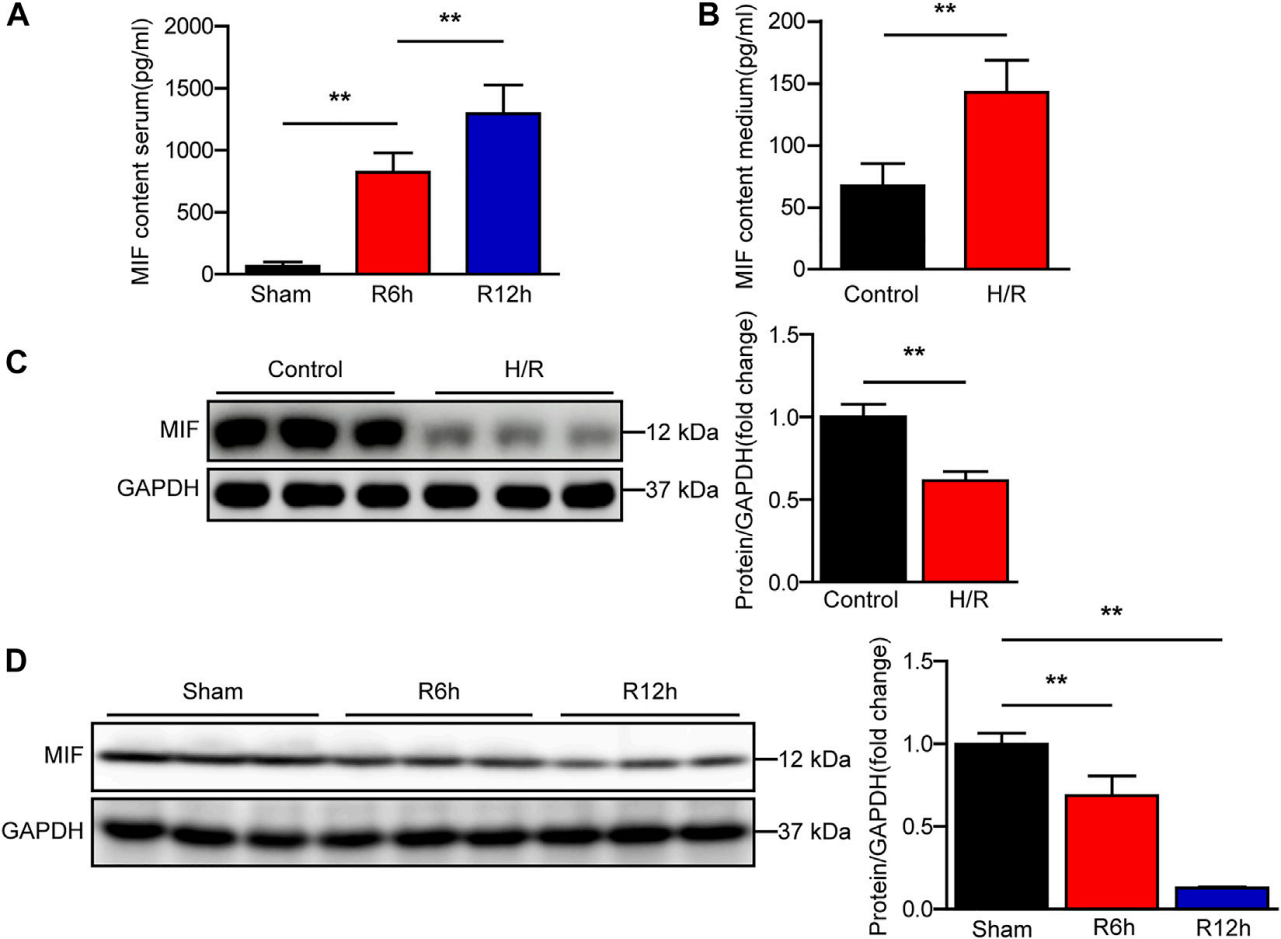
MIF expression is significantly increased in serum of mice subjected to hepatic I/R injury. **(A)** ELISA detection of MIF level in serum of mice at 6 and 12 h after hepatic IR surgery (*n* = 6 per group). **(B)** ELISA detection of MIF level in medium of hepatocytes subjected to H/R treatment (*n* = 3 per group). **(C)** MIF protein expression in cultured hepatocytes challenged by H/R treatment. GAPDH served as the loading control. Representative of three independent experiments. **(D)** MIF protein expression in the liver of mice at 6 and 12 h after hepatic IR surgery (n = 6 per group). GAPDH served as the loading control. All data are presented as the mean ± SD. Levels of statistical significance are indicated as **p < 0.01. For statistical analysis, one-way ANOVA with Bonferroni’s posthoc analysis or Tamhane’s T2 posthoc analysis and two-tailed Student’s t test were used.

To analyze the correlation between MIF and liver I/R injury, we assessed the expression of MIF after reperfusion in our mouse and cell model. Using ELISA, we found that MIF levels in mice serum and culture medium of AML12 hepatocytes was markedly increased after I/R or H/R treatment (Figures 1A,B). We further evaluated MIF protein expression in liver tissues after I/R injury and AML12 hepatocytes challenged by H/R using western blotting. Interestingly, we found that MIF protein expression was significantly downregulated *in vivo* and *in vitro* (Figures 1C,D). We speculate that MIF is released into the serum or medium after the hepatocytes are stimulated by I/R or H/R. Taken together, these data suggest that MIF participates in the regulation of hepatic I/R injury.

### MIF exacerbates liver damage after hepatic I/R injury

**FIGURE 2:**
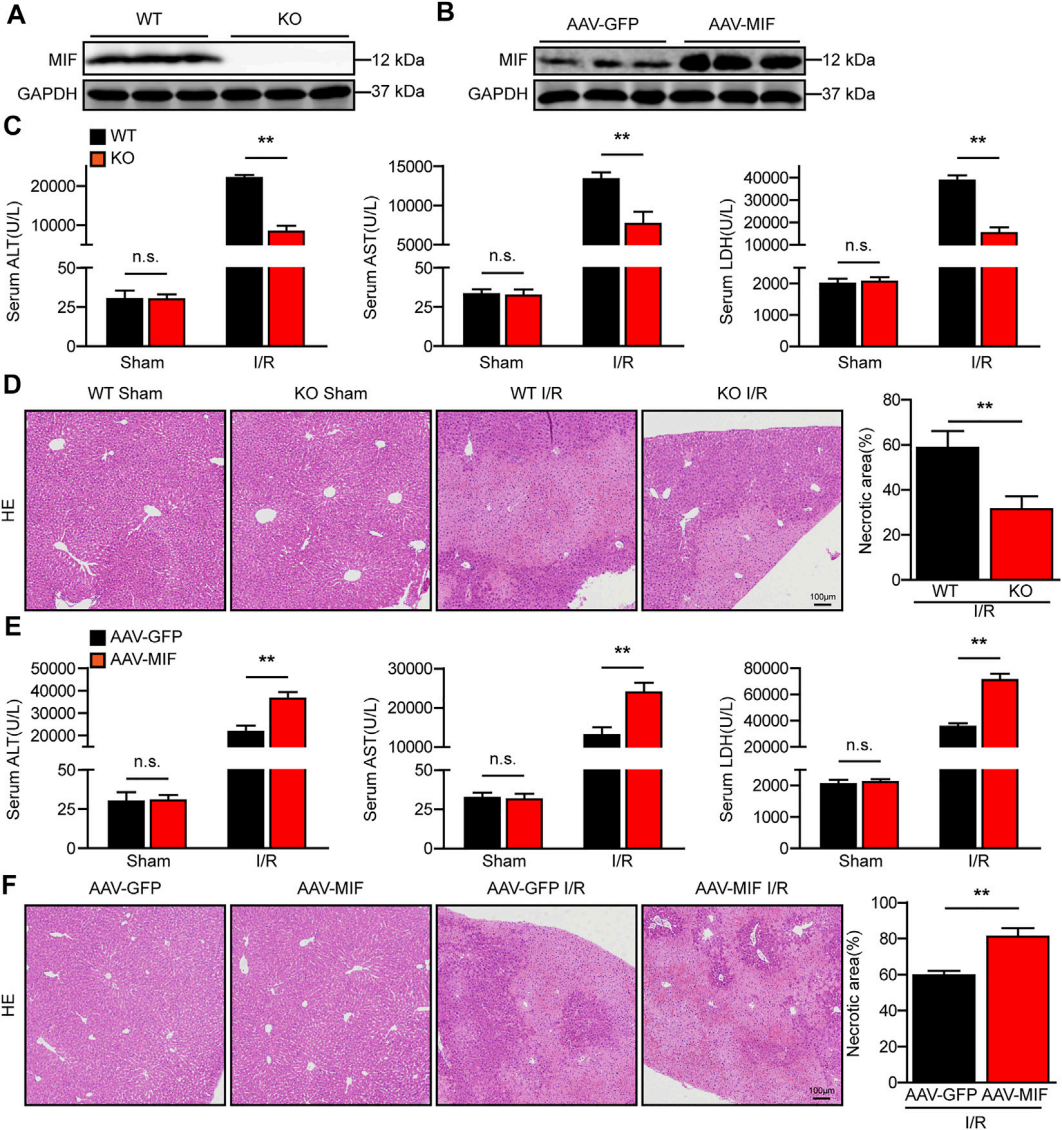
MIF deficiency alleviates liver damage after hepatic I/R injury. **(A)** MIF protein expression in the liver of WT and MIF-KO mice. GAPDH served as the loading control (*n* = 3 per group). **(B)** MIF protein expression in the liver of mice injected with AAV-GFP or AAV-MIF. GAPDH served as the loading control (*n* = 3 per group). **(C)** Serum ALT/AST/LDH activities in WT and MIF-KO mice after hepatic IR surgery (n = 6/group). **(D)** Representative histological HE-stained images and statistics showing necrotic areas in liver tissue from WT and MIF-KO mice after hepatic IR surgery (*n* = 6/group). Scale bar, 100 μm. **(E)** Serum ALT/AST/LDH activities in the serum of AAV-GFP or AAV-MIF mice after hepatic IR injury (*n* = 6/group). **(F)** Representative histological HE-stained images and statistics showing necrotic areas in the liver of AAV-GFP or AAV-MIF mice after hepatic IR injury (*n* = 6/group). Scale bar, 100 μm. All data are presented as the mean ± SD. Levels of statistical significance are indicated as**p < 0.01 and n. s = not significant. For statistical analysis, one-way ANOVA with Bonferroni’s post hoc analysis or Tamhane’s T2 post hoc analysis and two-tailed Student t test were used.

We next investigated whether MIF mediates hepatic I/R injury using MIF KO mice and mice injected with AAV8 to deliver MIF expression. MIF deletion or overexpression in the liver was confirmed by western blot analyses (Figures 2A,B). Notably, MIF deletion significantly reduced the elevated serum levels of ALT and AST in mice subjected to liver I/R injury compared with levels in WT control mice (Figure 2C). Compared with the WT controls, considerably fewer necrosis was found in the liver sections of MIF-KO mice after hepatic I/R injury as assessed by hematoxylin and eosin staining (Figure 2D). We further confirmed the role of MIF overexpression in liver I/R injury. In contrast to MIF-KO mice, AAV-MIF mice exhibited the opposite phenotype. Mice with MIF overexpression showed increased levels of AST and ALT in serum and more necrosis in liver tissues compared with AAV-GFP control mice after hepatic I/R injury (Figures 2E,F). These results suggest that MIF deficiency protects against hepatic I/R injury.

### MIF promotes inflammatory response during hepatic I/R injury

**FIGURE 3:**
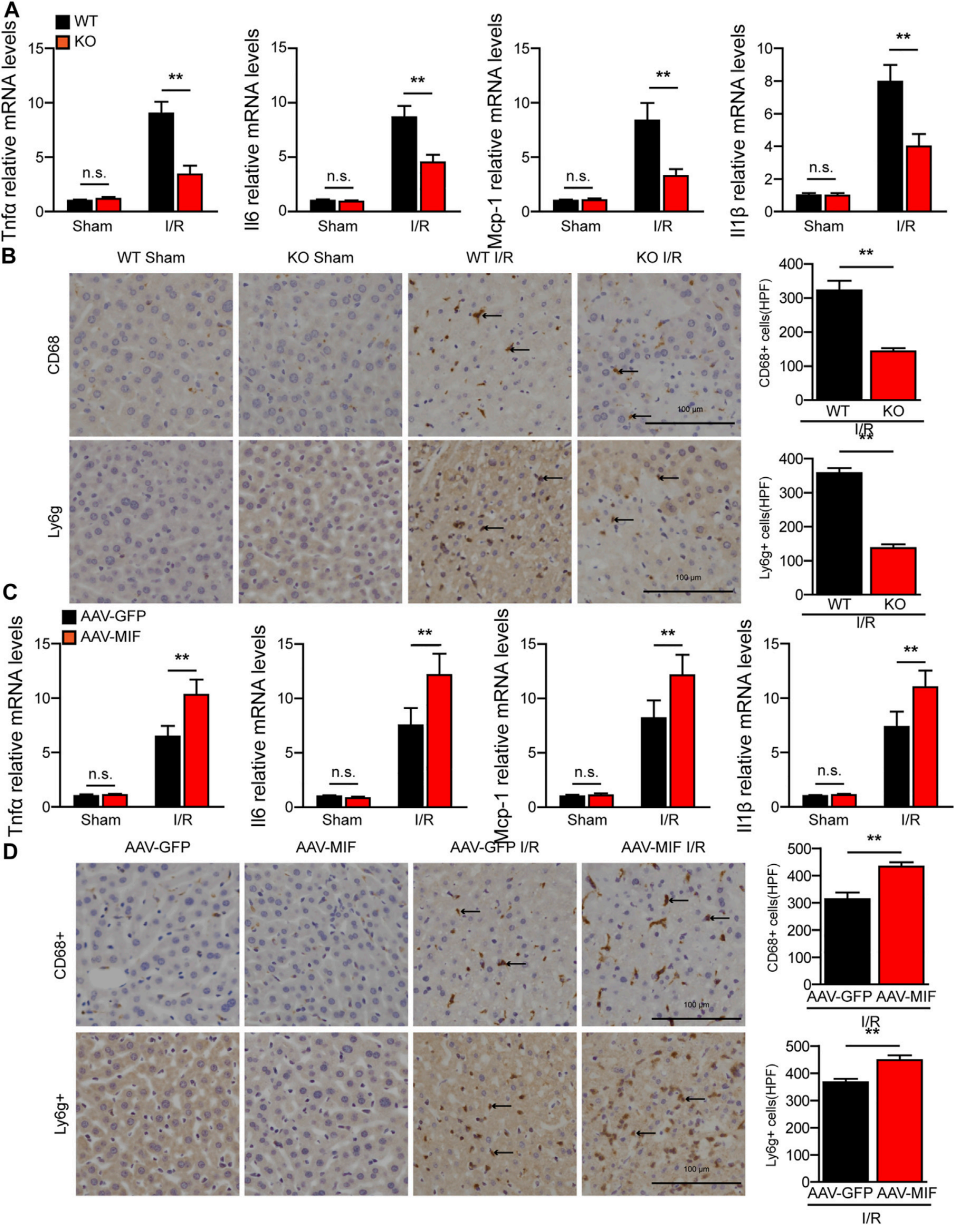
MIF promotes inflammatory response during hepatic I/R injury. **(A)** mRNA levels of proinflammatory factors (*Tnfα*, *Il6*, *IL1β*, and *Mcp-1*) in the liver of WT and *MIF*-KO mice after hepatic I/R surgery (*n* = 4/group). **(B)** Representative CD68 and Ly6g IHC staining in the liver lobes of WT and *MIF*-KO mice after hepatic I/R surgery (*n* = 4/group). Scale bar, 100 μm. Marked by black arrow. **(C)** mRNA levels of proinflammatory factors (*Tnfα*, *Il6*, *IL1β*, and *Mcp-1*) in the liver of AAV-GFP or AAV-MIF mice after hepatic I/R injury (*n* = 4/group). **(D)** Representative CD68 and Ly6g IHC staining in the liver of AAV-GFP or AAV-MIF mice after hepatic I/R injury (*n* = 4/group). Scale bar, 100 μm. Marked by black arrow. All data are presented as the mean ± SD. Levels of statistical significance are indicated as**p < 0.01 and n. s = not significant. For statistical analysis, one-way ANOVA with Bonferroni’s post hoc analysis or Tamhane’s T2 post hoc analysis and two-tailed Student t test were used.

During hepatic I/R injury, the inflammatory response plays a pivotal role throughout the ischemia and reperfusion phases and inhibition of inflammation can effectively alleviate liver I/R injury (Zhang et al., 2019). As a proinflammatory cytokine, we tested the role of MIF in the inflammatory response in hepatic I/R injury. As shown in Figure 3A, MIF deficiency significantly reduced gene expression of inflammatory factors such as tumor necrosis factor a (TNFα), interleukin 6 (IL6), interleukin 1b (IL1β), and monocyte chemoattractant protein-1 (MCP-1) in the liver of mice subjected to I/R injury compared with the WT controls. Moreover, after liver I/R surgery, immunohistochemistry staining revealed that the number of inflammatory immune cells (CD68^+^ monocyte-macrophages and Ly6g + neutrophils) in the liver of MIF-KO mice was significantly decreased compared with those of their counterparts (Figure 3B). In contrast, AAV-MIF mice showed significantly higher expression of inflammatory cytokines and more inflammatory-cell infiltration in the liver of mice challenged by I/R (measured by qRT-PCR and IHC staining) compared with AAV-GFP mice (Figures 3C,D). These experiments suggest that MIF is an essential factor controlling the inflammatory response during liver I/R injury.

### MIF regulates apoptosis in hepatic I/R injury

**FIGURE 4:**
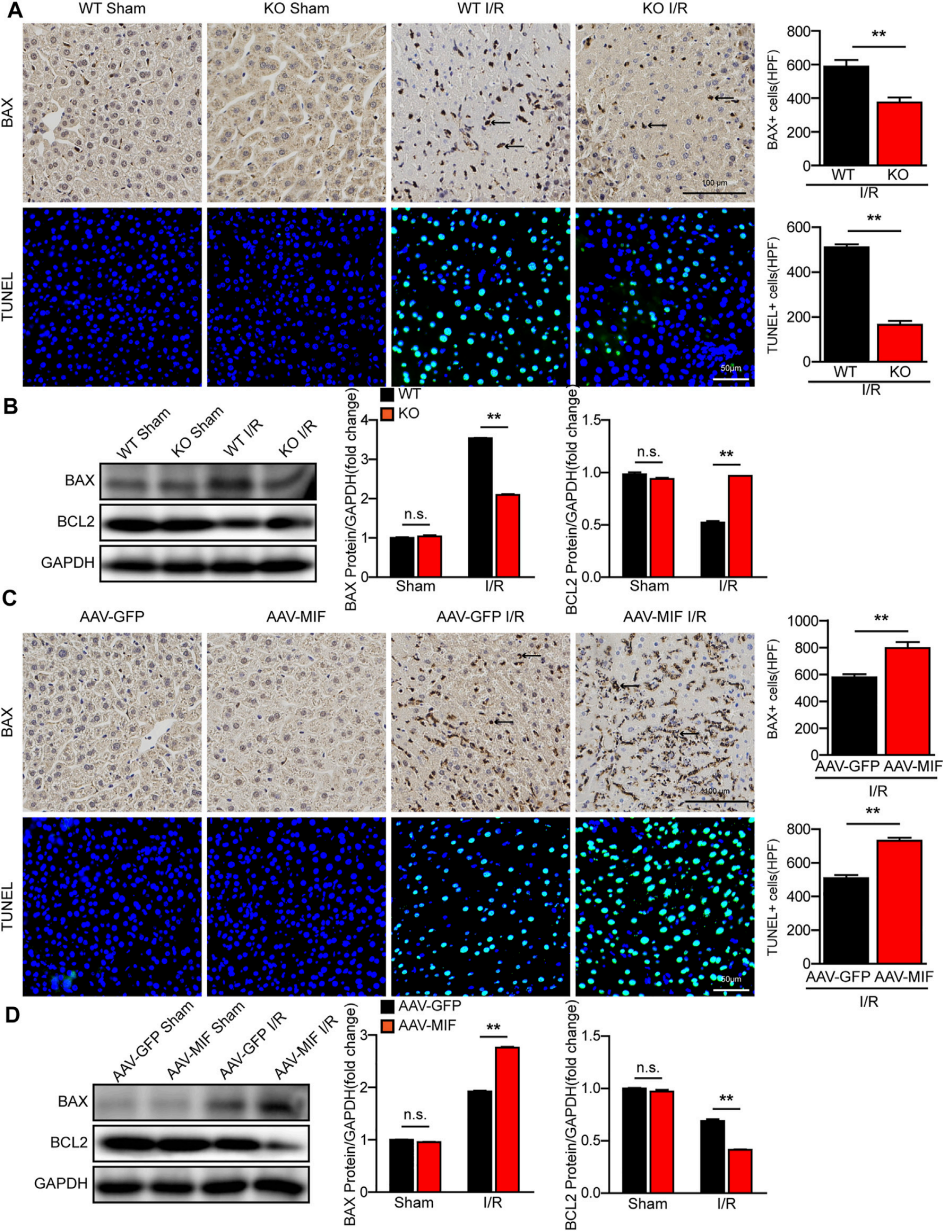
MIF deletion suppresses apoptosis in hepatic I/R injury. **(A)** IHC staining of BAX and TUNEL staining in liver sections from WT and MIF-KO mice after hepatic I/R surgery (*n* = 4/group). Scale bar, 100 μm of IHC staining and 50 μm of TUNEL staining respectively. Marked by black arrow. **(B)** Western blot analysis of apoptosis-related molecules (BAX, BCL2 and C-Caspase-3) protein levels in the liver of WT and MIF-KO mice at 6 h after hepatic I/R surgery. GAPDH served as a loading control (*n* = 3/group). **(C)** IHC staining of BAX and TUNEL staining in liver sections from AAV-GFP or AAV-MIF mice after hepatic I/R injury (*n* = 4/group). Scale bar, 100 μm of IHC staining and 50 μm of TUNEL staining respectively. Marked by black arrow. **(D)** Western blot analysis of apoptosis-related molecules (BAX, BCL2 and C-Caspase-3) protein levels in the liver of AAV-GFP or AAV-MIF mice after hepatic I/R injury. GAPDH served as a loading control (*n* = 3/group). All data are shown as the mean ± SD. Levels of statistical significance are indicated as **p < 0.01. For statistical analysis, one-way ANOVA with Bonferroni’s post hoc analysis or Tamhane’s T2 post hoc analysis and two-tailed Student t test were used.

Cellular apoptosis is directly involved in liver damage during hepatic I/R injury (Zheng et al., 2020). In our study, IHC and TUNEL staining revealed that the number of apoptotic cells significantly increased in WT mice after hepatic I/R injury, while MIF deletion significantly suppressed hepatocyte apoptosis in liver tissues (Figure 4A). Furthermore, the expression of c-caspase 3 and the ratio between the pro-apoptotic protein Bax and the anti-apoptotic protein Bcl-2 were also dramatically reduced in the MIF-KO mice subjected to I/R injury (Figure 4B). In contrast, MIF overexpression aggravated apoptosis during hepatic I/R injury compared with the control mice, as demonstrated by induction of Bax and TUNEL-positive nuclei, a higher BAX/Bcl-2 ratio, and upregulation levels of c-caspase 3 (Figures 4C,D). Collectively, these data suggest that MIF deficiency suppresses liver apoptosis during hepatic I/R injury.

### MIF aggravates oxidative stress in hepatic I/R injury

**FIGURE 5:**
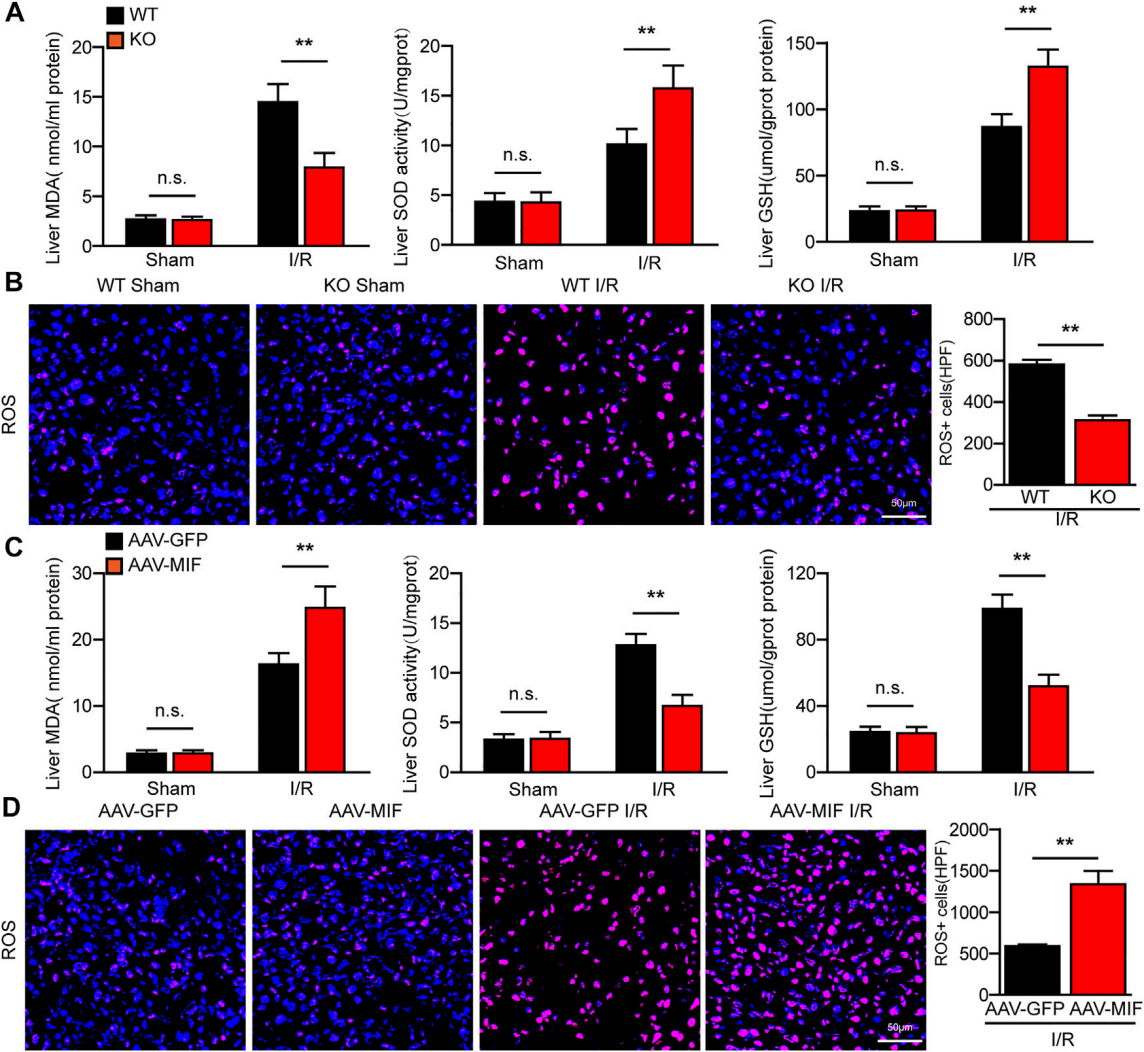
MIF ablation inhibits oxidative stress in hepatic I/R injury. **(A)** ELISA detection of MDA, SOD and GSH level in serum of WT and MIF-KO mice after hepatic I/R surgery (*n* = 6 per group). **(B)** DHE staining in liver sections from WT and MIF-KO mice after hepatic I/R surgery (*n* = 4/group). Scale bar, 50 μm. **(C)** ELISA detection of MDA, SOD and GSH level in serum of AAV-GFP or AAV-MIF mice after hepatic I/R surgery (*n* = 6 per group). **(D)** DHE staining in liver sections from AAV-GFP or AAV-MIF mice after hepatic I/R surgery (*n* = 4/group). Scale bar, 50 μm. All data are shown as the mean ± SD. Levels of statistical significance are indicated as **p < 0.01. For statistical analysis, one-way ANOVA with Bonferroni’s post hoc analysis or Tamhane’s T2 post hoc analysis and two-tailed Student t test were used.

During hepatic I/R injury, the accumulation of ROS promotes several cellular processes that aggravate liver damage, including metabolism disequilibrium, apoptosis, and inflammation (Bi et al., 2019). Interestingly, we found that the level of oxidative related factor MDA was significantly reduced, whereas the anti-oxidative factors SOD and GSH were markedly increased in the liver of MIF-KO mice subjected to hepatic I/R surgery compared with those in WT control mice (Figure 5A). Meanwhile, DHE staining showed that ROS production was highly suppressed by MIF deficiency after I/R injury (Figure 5B). However, AAV-mediated MIF overexpression exacerbates oxidative stress in the livers of mice after hepatic I/R injury compared with the AAV-GFP control mice, as demonstrated by higher levels of MDA and ROS and lower levels of SOD and GSH in liver (Figures 5C,D). These findings indicate that MIF ablation inhibits oxidative stress in hepatic I/R injury.

### MIF knock-down protects hepatocytes from H/R injury

**FIGURE 6:**
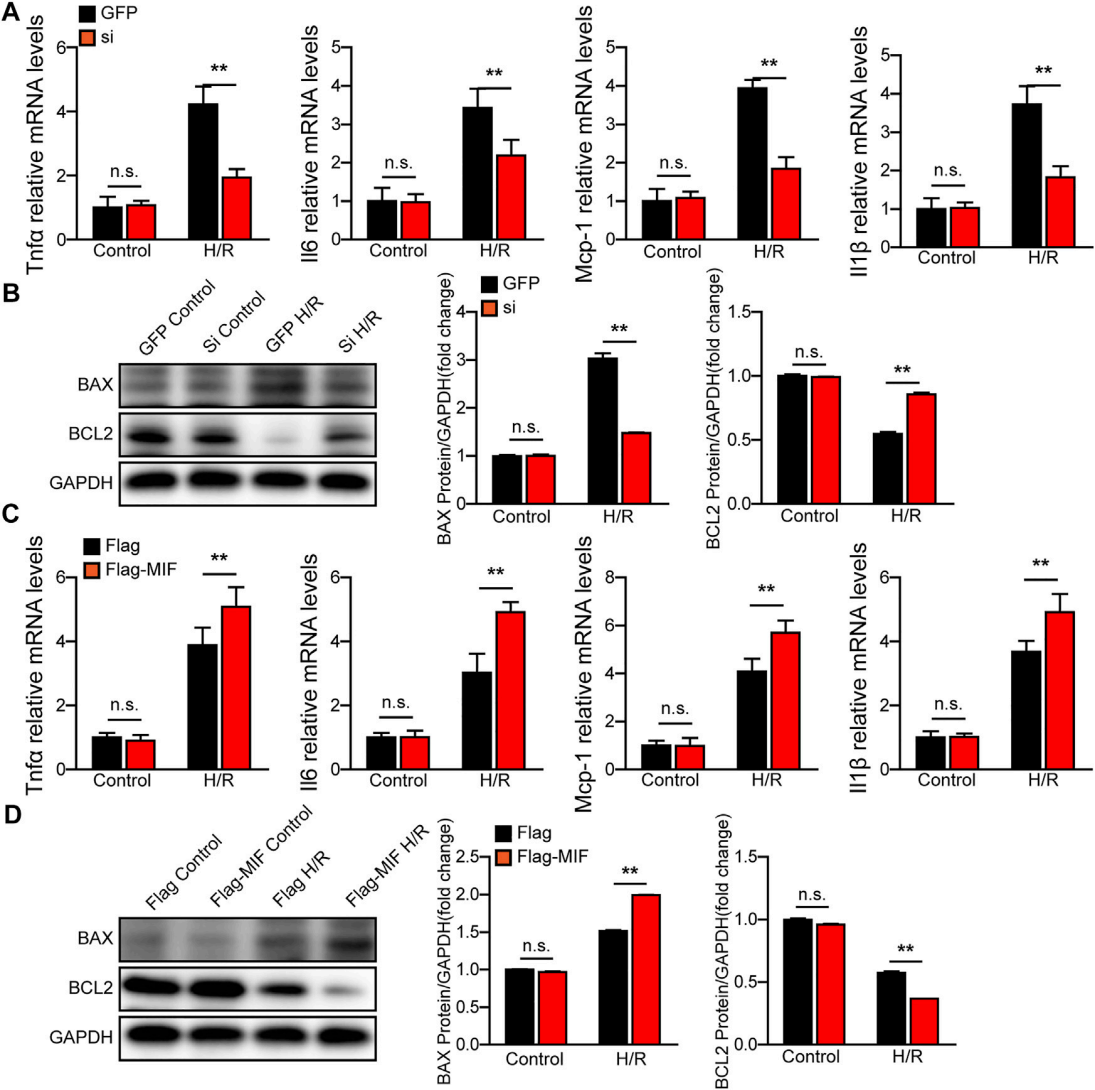
MIF knock-down protects hepatocytes from H/R injury. **(A)** The mRNA levels of proinflammatory factors (*Tnfα*, *Il6*, *IL1β*, and *Mcp-1*) in cultured MIF knockdown hepatocytes challenged by H/R treatment. Representative of three independent experiments. **(B)** Western blot analysis of the protein expression levels of Caspase3, Bax, and Bcl2 in cultured MIF knockdown hepatocytes challenged by H/R treatment. GAPDH served as the loading control. Representative of three independent experiments. **(C)** The mRNA levels of proinflammatory factors (*Tnfα*, *Il6*, *IL1β*, and *Mcp-1*) in cultured MIF overexpressed hepatocytes challenged by H/R treatment. Representative of three independent experiments. **(D)** Western blot analysis of the protein expression levels of Caspase3, Bax, and Bcl2 in cultured MIF overexpressed hepatocytes challenged by H/R treatment. GAPDH served as the loading control. Representative of three independent experiments. All data are shown as the mean ± SD. Levels of statistical significance are indicated as **p < 0.01. For statistical analysis, one-way ANOVA with Bonferroni’s post hoc analysis or Tamhane’s T2 post hoc analysis were used.

We further evaluated the function of MIF in AML12 hepatocytes challenged by H/R stimulation. We used MIF siRNA and MIF expression plasmids to establish MIF-knockdown or MIF-overexpressing hepatocytes, respectively. Compared with the control group, MIF knockdown markedly inhibited hepatocyte inflammation, apoptosis and oxidative stress after H/R challenge, as determined by RT-PCR detecting inflammatory cytokines (TNFα, IL6, IL1β and MCP-1) (Figure 6A), ELISA detecting oxidative stress factors (MDA, SOD and GSH) (Supplementary Figure S6A), and western blot detecting c-caspase3, BAX, and Bcl-2 (Figure 6B). However, hepatocytes transfected with Flag-MIF exhibited exacerbated inflammation, apoptosis and oxidative stress after H/R stimulation compared with those in Flag Controls (Figures 6C,D and Supplementary Figure S6B). These findings suggest that MIF knock protects against H/R-induced injury in hepatocytes.

### MIF activates the ASK1-JNK/p38 signaling during hepatic I/R injury

**FIGURE 7:**
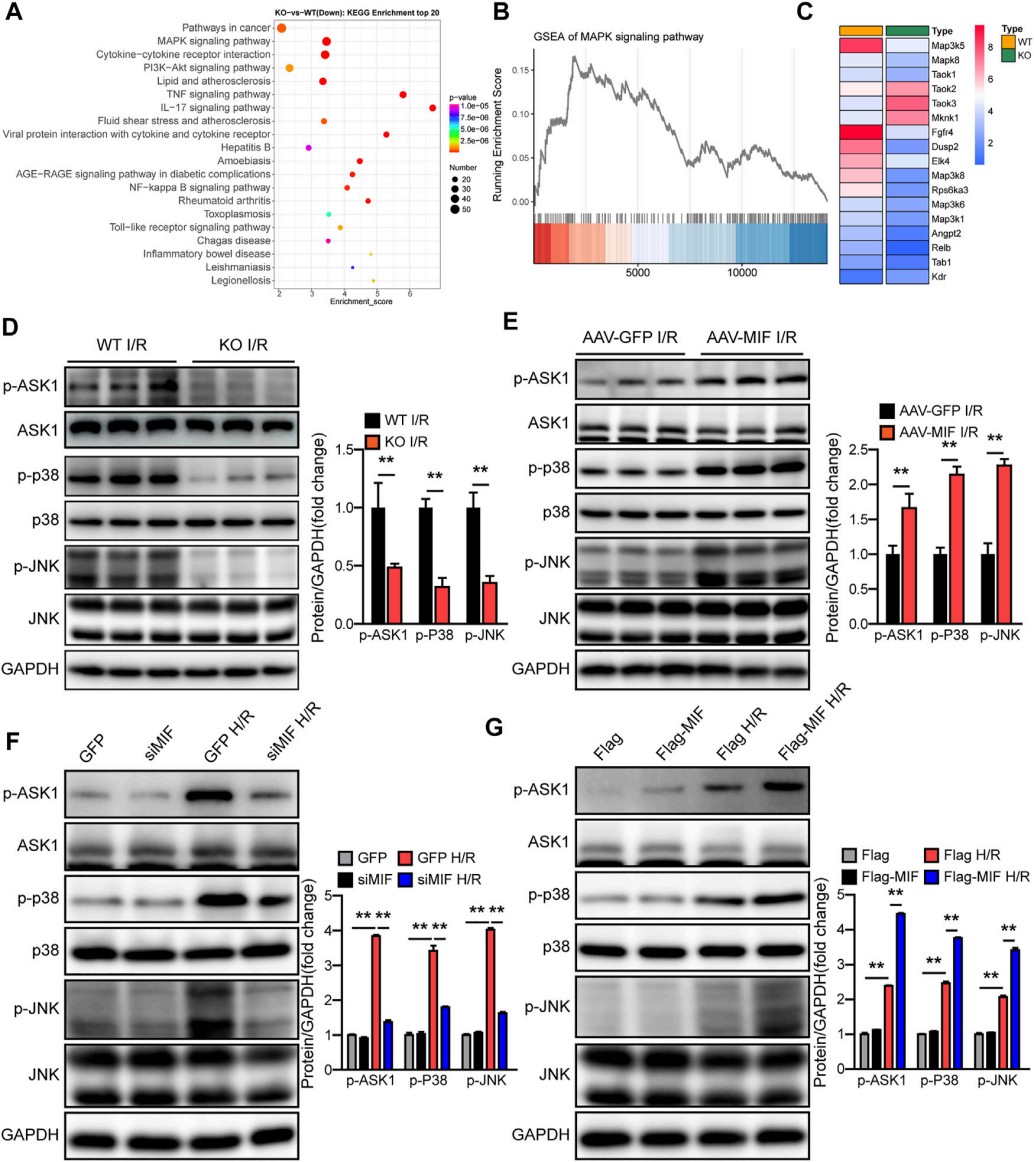
MIF activates the ASK1-JNK/p38 signaling during hepatic I/R injury. **(A)** KEGG pathway enrichment analysis of the major biological pathways contributing to MIF function. **(B)** GSEA-enriched analysis of MAPK pathways. **(C)** Heatmap showing expression of genes involved in MAPK pathways. **(D)** Protein levels of the total and phosphorylated protein expression levels of ASK1, P38, and JNK in the liver of MIF-KO and WT mice after hepatic I/R operation. GAPDH served as the loading control (*n* = 3 per group). **(E)** Protein levels of the total and phosphorylated protein expression levels of ASK1, P38, and JNK in the liver of AAV-GFP and AAV-MIF mice after hepatic I/R operation mice. GAPDH served as the loading control (*n* = 3 per group). **(F)** Protein levels of the total and phosphorylated protein expression levels of ASK1, P38, and JNK in GFP and siMIF hepatocytes that were challenged by H/R insult. GAPDH served as the loading control. Representative of three independent experiments. **(G)** Protein levels of the total and phosphorylated protein expression levels of ASK1, P38, and JNK in Flag and Flag-MIF hepatocytes that were challenged by H/R insult. GAPDH served as the loading control. Representative of three independent experiments. All data are presented as the mean ± SD. Levels of statistical significance are indicated as**p < 0.01. For statistical analysis, two-tailed Student t test were used.

To understand the underlying mechanisms responsible for the positive effect of MIF on liver I/R injury, we performed RNA sequencing (RNA-Seq) with I/R-challenged liver samples of WT and MIF-KO mice. Principal component analysis (PCA) defined the primary determinants of differences between liver samples of WT and MIF-KO mice (PC1, 38.1%; PC2, 17.7%). All samples were clearly separated into their respective groups (Supplementary Figure S7). Kyoto Encyclopedia of Genes and Genomes (KEGG) pathway enrichment analysis showed that the MAPK signaling pathway was one of the most significantly down regulated enriched pathways contributing to MIF-mediated hepatic I/R injury (Figure 7A). Gene Set Enrichment Analysis (GSEA) analysis indicated that MAPK pathway was down regulated in MIF-KO mice during hepatic I/R injury (Figure 7B). Heat map analysis showed that ASK1 (MAP3K5) was also significantly down regulated in MIF-KO mice during hepatic I/R injury (Figure 7C). We further confirmed the influence of MIF on MAPK signaling by loss- and gain-of-function of MIF *in vivo* and *in vitro*. In line with the results from RNA-seq, MIF deficiency suppressed ASK1, JNK and p38 phosphorylation post hepatic I/R injury and hepatocyte challenged by H/R treatment (Figures 7D,F), while MIF overexpression significantly promoted the ASK1, JNK and p38 phosphorylation compared to control group (Figures 7E,G). Together, these results suggest that MIF activates the ASK1-JNK/P38 signaling during hepatic I/R injury.

### The protective effect of MIF deficiency in hepatic I/R injury depends on the suppression of ASK1

**FIGURE 8:**
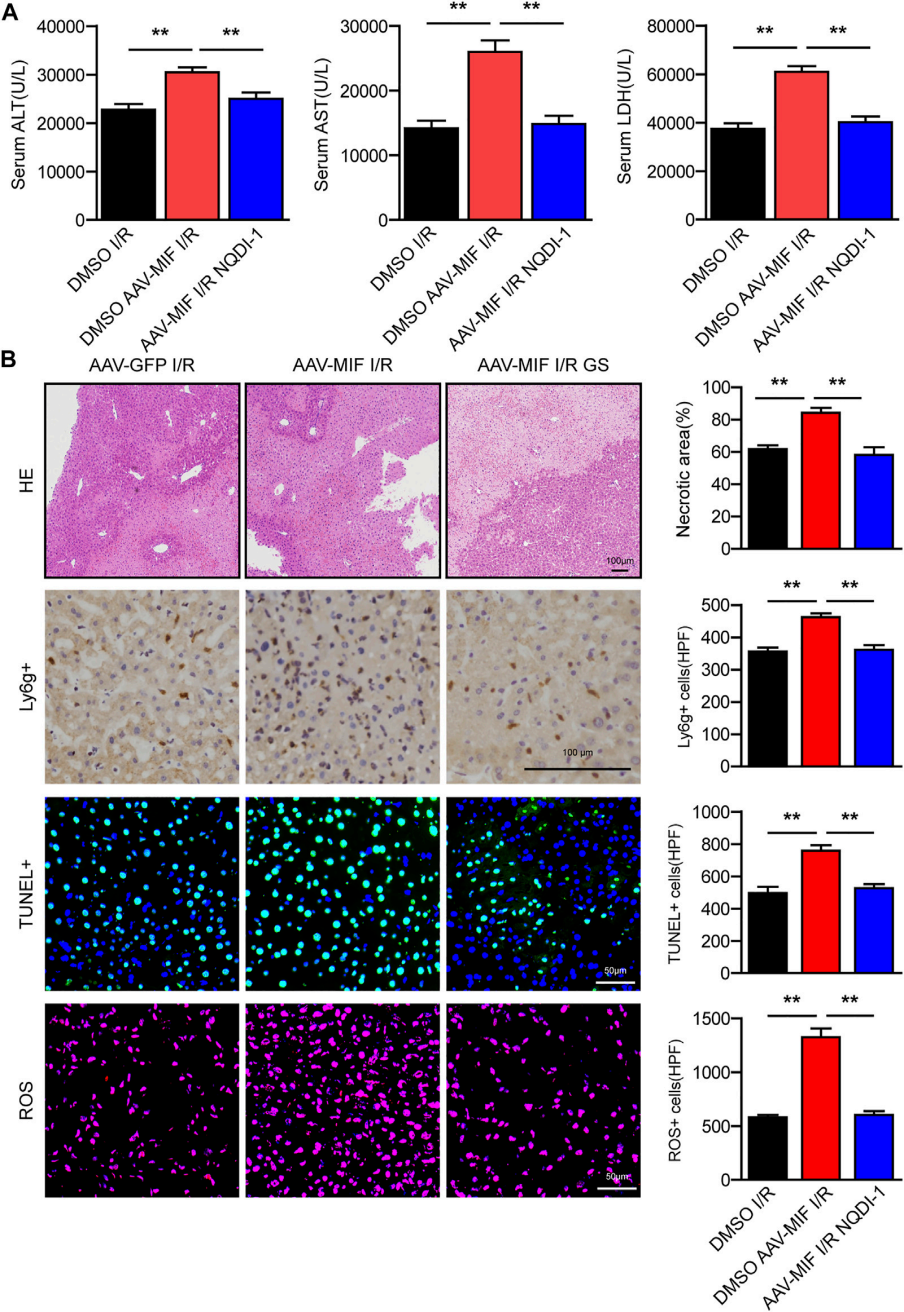
The protective effect of MIF deficiency in hepatic I/R injury depends on the suppression of ASK1. **(A)** Serum ALT/AST/LDH activities in mice after hepatic I/R surgery (*n* = 6/group). **(B)** Representative histological HE-stained images, Ly6g positive IHC stained images, TUNEL stained images, DHE stained images and statistical analysis in liver tissue of mice after hepatic I/R surgery (*n* = 4–6/group). Scale bar, 100 μm. All data are presented as the mean ± SD. Levels of statistical significance are indicated as**p < 0.01. For statistical analysis, one-way ANOVA with Bonferroni’s post hoc analysis or Tamhane’s T2 post hoc analysis.

To ascertain the critical role of ASK1-JNK/P38 pathway in MIF-mediated hepatic I/R injury, we used a specific ASK1 inhibitor (NQDI-1) to perform an intraperitoneal injection in AAV-MIF mice prior to I/R injury. Compared with I/R group, NQDI-1 largely abolished the MIF overexpression-induced enhancement of liver injury, as demonstrated by reduced serum aminotransferase activity (ALT, AST, LDH) and decreased liver necrosis, inflammation, apoptosis, and oxidative stress in mice challenged by hepatic I/R injury (Figures 8A,B). Thus, it can be deduced that the protective effect of MIF deficiency in hepatic I/R injury depends on the suppression of ASK1.

### Pharmacological inhibition of MIF attenuated I/R responsive liver injury

**FIGURE 9:**
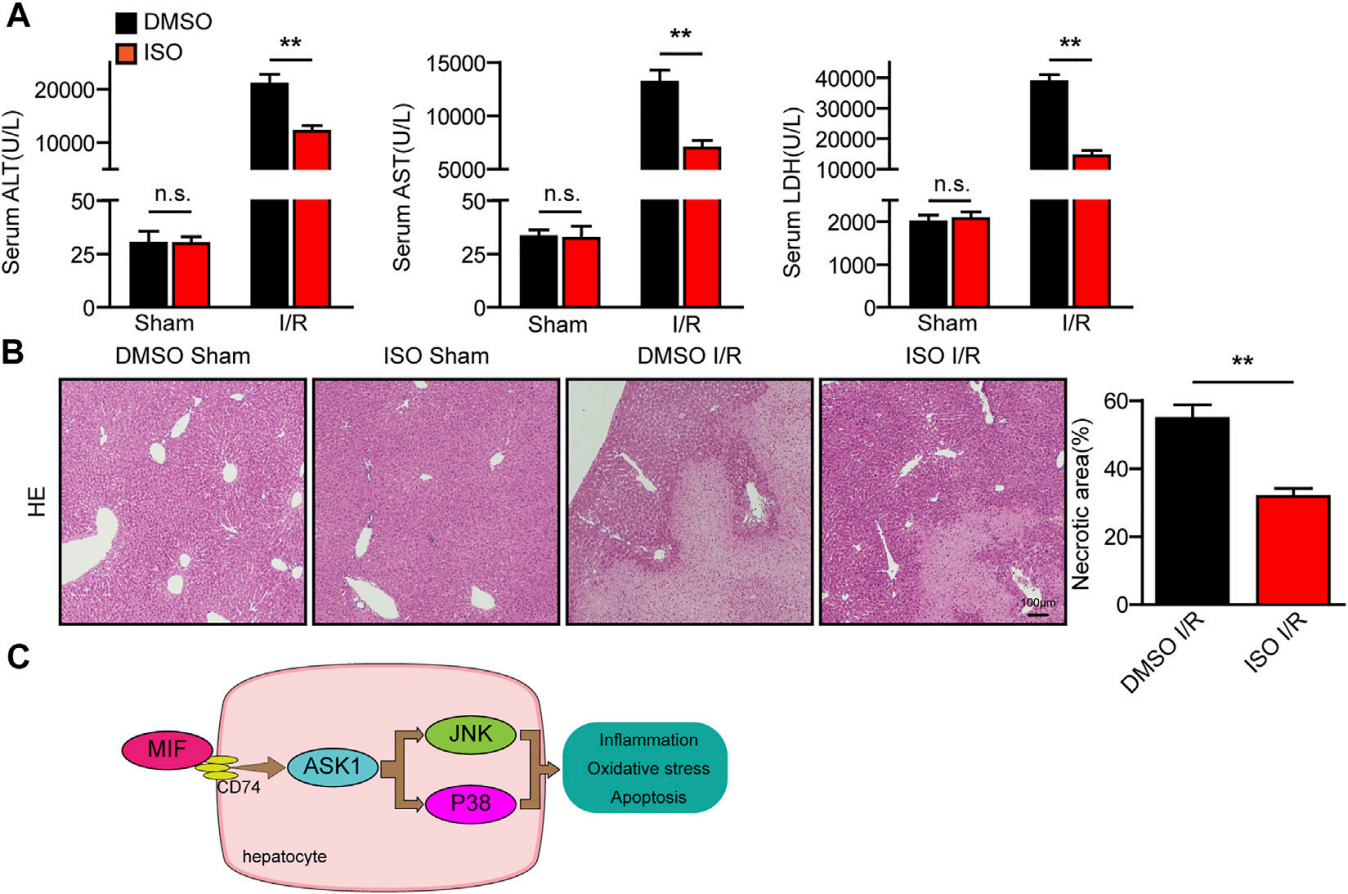
Pharmacological inhibition of MIF attenuated the I/R responsive liver injury. **(A)** Serum ALT/AST/LDH activities in mice after hepatic I/R surgery (*n* = 6/group). **(B)** Representative histological HE-stained images and statistics showing necrotic areas in liver tissue of mice after hepatic I/R surgery (*n* = 6/group). Scale bar, 100 μm. **(C)** Mechanism of MIF regulating liver I/R injury. All data are presented as the mean ± SD. Levels of statistical significance are indicated as**p < 0.01. For statistical analysis, one-way ANOVA with Bonferroni’s post hoc analysis or Tamhane’s T2 post hoc analysis.

Finally, we used a specific inhibitor of MIF (ISO-1) to explore its therapeutic effect on hepatic I/R injury. As shown in Figure 9A, ISO-1 treated mice exhibited lower ALT and AST activities than those of DMSO treated mice after liver I/R surgery. Furthermore, histological analysis of the livers also demonstrated reduced necrotic area in ISO-1 treated mice compared with that of DMSO treated mice (Figure 9B). These results suggest that pharmacological inhibition of MIF attenuated I/R responsive liver injury and MIF could therefore serve as a potential therapeutic strategy for treating hepatic injury.

## Discussion

Hepatic I/R injury leads to irreversible liver damage, but the mechanism has not been fully resolved. MIF is abundantly expressed in various tissues and regulates multiple biological processes (Osipyan et al., 2021). MIF has been reported to be a key mediator in alcohol-induced liver injury by promoting inflammation (Marin et al., 2017). Paradoxically, other study reported that MIF improve myocardial infarction by suppressing oxidative stress and apoptosis (Liu et al., 2020). We speculate that this may be because MIF plays different roles in different diseases and organs. To date, the role and molecular mechanism of MIF in hepatic I/R injury remain unclear. In the present study, we revealed that MIF act as novel biomarker and therapeutic target for the diagnosis and treatment of hepatic I/R injury. During liver I/R injury, MIF was released from the liver into the serum. Functional experiments demonstrated that MIF deficiency significantly diminished I/R- or H/R-induced inflammation, apoptosis, and oxidative stress in both cultured hepatocytes and mice. Moreover, we suggest that these beneficial effects of MIF deletion on hepatic I/R injury are largely dependent on the suppression of the ASK1-JNK/P38 pathway (Figure 9C).

MIF can not only promote the activation of T cells and macrophages, but also promote the chemical attraction to immune cells, promote the production of inflammatory cytokines and stress molecules, and resist the immunosuppressive effect of glucocorticoids. Therefore, MIF is involved in the pathogenesis of many autoimmune and inflammatory diseases, including rheumatoid arthritis (RA), liver injury, and pancreatitis. Moreover, it has been reported that MIF acts in an autocrine and paracrine manner by binding to receptors CD74/CD44, CXCR2, CXCR4 and CXCR7, thereby activating downstream MAPK, AMPK and Akt signals to mediate pro-inflammatory responses (Kong YZ, et al. Int J Mol Sci. 2022 April 28; 23 (9):4908; Farr L, et al. Front Immunol. 2020 June 23; 11:1273.). In the process of liver I/R injury, we speculate that MIF from macrophages and hepatocytes aggravates liver injury by promoting the production of inflammatory chemokines and promoting inflammatory response by activating MAPK signaling pathway.

Extensive research has shown that inhibition of the inflammatory response may be an effective strategy to ameliorate hepatic I/R injury (Yan et al., 2019). During hepatic I/R injury, macrophages and neutrophils are recruited to the ischemic liver, contributing to ROS production and hepatocellular apoptosis (Abu-Amara et al., 2010). MIF can not only promote the activation of T cells and macrophages, but also promote the chemical attraction to immune cells, promote the production of inflammatory cytokines and stress molecules, and resist the immunosuppressive effect of glucocorticoids. Moreover, it has been reported that MIF acts in an autocrine and paracrine manner by binding to receptors CD74/CD44, CXCR2, CXCR4 and CXCR7, thereby activating downstream MAPK, AMPK and Akt signals to mediate pro-inflammatory responses (Farr et al., 2020; Kong et al., 2022). As a pro-inflammatory cytokine, the association between MIF and the inflammatory response has been reported in different studies and is the subject of some controversy. It seems that MIF might either positively or negatively regulate the inflammatory response. In our previous study using a mouse model of severe acute pancreatitis, we found that deletion of MIF reduced inflammation (Zhu et al., 2020). However, other research suggests that MIF serves as a defense mechanism, decreasing hepatic inflammatory cells and pro-inflammatory cytokines in nonalcoholic steatohepatitis (Heinrichs et al., 2014). In the present study, we found that MIF deteriorated the inflammatory response during hepatic I/R injury *in vivo* and *in vitro*. We speculate that MIF from macrophages and hepatocytes aggravates liver injury by promoting the production of inflammatory chemokines and promoting inflammatory response by activating MAPK signaling pathway.

During hepatic I/R injury, anoxia followed by reperfusion results in massive ROS production, inducing activation of inflammatory cells and cell death (Li et al., 2015). MIF has been shown to promote apoptosis and oxidative stress in many diseases (Yang et al., 2020; Wang et al., 2021). Studies have indicated that MIF deficiency improve hepatic function by suppressing oxidative stress and inflammation in thioacetamide-induced liver Injury in mice (Vukićević et al., 2021). Moreover, MIF inhibition significantly decreases the production of nitric oxide synthase (iNOS) as well as ROS in LPS-stimulated microglia cells (Zhang et al., 2016). However, it has also been reported that MIF deletion abolished the protective effects of ischemic preconditioning in myocardial I/R injury through aggravating myocyte apoptosis, ROS production, inflammatory-cell infiltration (Ruze et al., 2019). In the present study, our *in vivo* and *in vitro* results also showed that MIF exacerbated the apoptosis and oxidative stress in hepatic I/R injury.

MAPK signaling has been recognized as the major molecular event contributing to hepatic I/R injury (Imarisio et al., 2017; Wang et al., 2017). During the hepatic I/R process, damage-associated molecular patterns (DAMPs) and ROS stimulation of hepatocytes can activate JNK and p38 signals that result in cellular inflammation and apoptosis (Yang et al., 2019; Zhou et al., 2021b). Importantly, MIF has been reported to function in regulating MAPK signaling. Previous studies have demonstrated that MIF promotes MAPK signaling through activation or inhibition of JNK phosphorylation, depending on the cell type and underlying stimulation context (Lue et al., 2011). In addition, MIF inhibitor ISO-1 was reported to protect severe acute pancreatitis by suppressing phosphorylated P38 and nuclear factor-κB (NF-κB) signaling (Wang et al., 2020). In the present study, we found MAPK signaling was significantly activated during hepatic I/R injury. Further *in vivo* and *in vitro* experiments showed that MIF promoted the activation of JNK and P38 pathway. However, how MIF fine-tunes the inhibition of MARK signaling remains unknown. ASK1 acts as a key mediator of JNK/P38 activation, mediating cellular death and inflammatory responses in a cell- and stimulus-dependent manner (Ogier et al., 2020; Obsilova et al., 2021). More importantly, we found that the protective effect of MIF deficiency in hepatic I/R injury depends on the suppression of ASK1.

## Conclusion

In summary, the present study demonstrates that MIF deficiency protects from liver I/R injury via suppression of the inflammatory response, apoptosis, and oxidative stress. Moreover, the protective effect of MIF deficiency in hepatic I/R injury depends on the activation of ASK1-JNK/P38 signaling. These findings provide experimental evidence that may support MIF as a potential therapeutic target for strategies against hepatic I/R injury.

## Data Availability

The datasets presented in this study can be found in online repositories. The names of the repository/repositories and accession number(s) can be found in the Supplementary Material. The raw data of gene sequencing can be viewed at https://www.ncbi.nlm.nih.gov/geo/query/acc.cgi?acc=GSE212508 with number GSE212508.
