# Supplementary material for: Inhibition of macrophage migration inhibitory factor (MIF) suppresses apoptosis signal-regulating kinase 1 to protect against liver ischemia/reperfusion injury
Source: Front Pharmacol. 2022 Sep 8;13:951906. doi: 10.3389/fphar.2022.951906 (PMC9493190; doi:10.3389/fphar.2022.951906)
Supplement: Supplementary file 1 [file DataSheet1.PDF]

Supplementary data for:

Inhibition of macrophage migration inhibitory factor (MIF) suppresses apoptosis signal-regulating kinase 1 to protect against liver ischemia/reperfusion injury

Sanyang Chen[1,2,4†], Qiwen Yu[3,4†], Yaodong Song[1,2], Zongchao Cui[1,2], Mengke Li[1,2], Chaopeng Mei[1,2], Huning Cui[1,2], Shengli Cao[3,4*], Changju Zhu[1,2*]

1.Department of Emergency surgery, First Affiliated Hospital of Zhengzhou University, Zhengzhou, Henan, China

2.Henan Medical Key Laboratory of Emergency and Trauma Research

3.Department of Hepatobiliary and Pancreatic Surgery, First Affiliated Hospital of Zhengzhou University, Zhengzhou, Henan, China

4.Henan Key Laboratory of Digestive Organ Transplantation, Zhengzhou, Henan Province, 450052, China

[†]These authors contributed equally to this work.

Running Title: MIF deficiency protects from hepatic I/R injury.

***Corresponding authors.**

Sheng-Li Cao, M.D., Ph.D.

Department of Hepatobiliary and Pancreatic Surgery, First Affiliated Hospital of Zhengzhou University, Zhengzhou, Henan, China

Address: No.1, East Jian She Road, Zhengzhou, Henan Province, 450052

E-mail: shenglicao66@126.com

Chang-Ju Zhu, M.D., Ph.D.

Department of Emergency surgery, First Affiliated Hospital of Zhengzhou University, Zhengzhou, Henan, China

Address: No.1, East Jian She Road, Zhengzhou, Henan Province, 450052

E-mail: fccpanj@zzu.edu.cn

Supplementary Figures:

Supplementary Fig. 6

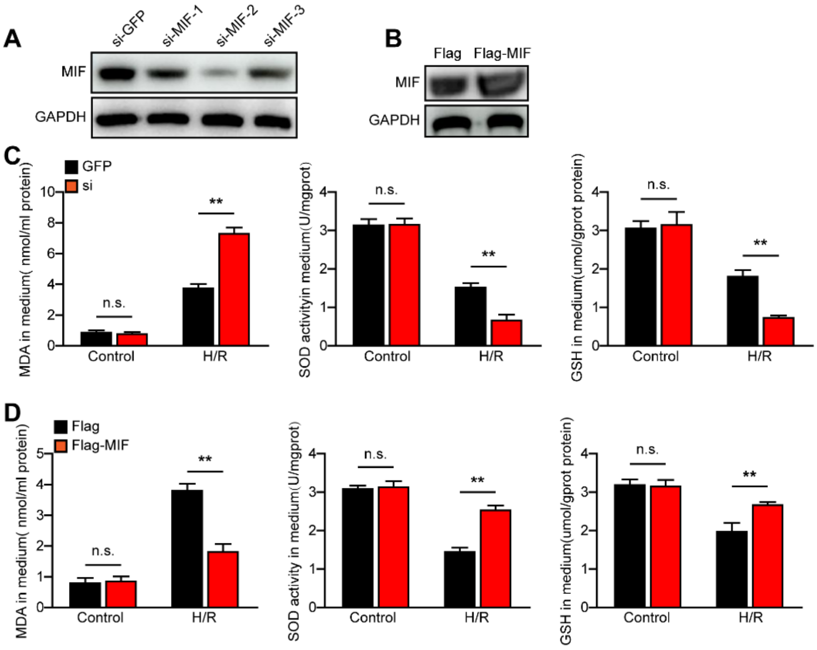

Fig. S6. (A) Western blot analysis of MIF in hepatocytes transfected by siRNA. GAPDH served as a loading control. (B) Western blot analysis of MIF in hepatocytes transfected by Flag-MIF. GAPDH served as a loading control. (C, D) ELISA detection of MDA, SOD and GSH level in the medium. All data are shown as the mean ± SD. Levels of statistical significance are indicated as ** $p < 0.01$.

Supplementary Fig. 7

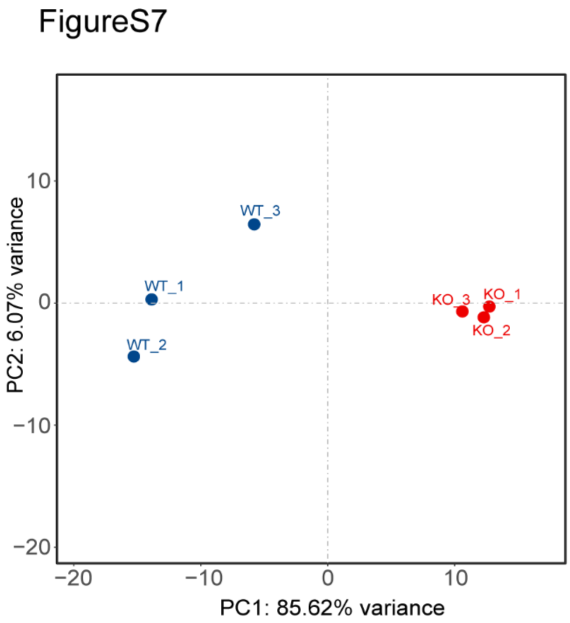

Fig. S7. PCA images showing global sample distribution profiles analyzed by

principal component analysis.

Supplementary Fig. 8

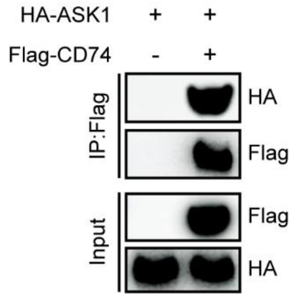

Fig. S8. Flag-tagged CD74 and HA-tagged ASK1 plasmids were co-transfected into HEK293T cells. Anti-Flag antibody was used for immunoprecipitation. Representative of three independent experiments.

Supplementary Tables

Supplementary Table 1. Primers for real-time PCR detection.

Gene	Specie		Sequence5'---3'
Tnfα	mice	F	CATCTTCTCAAAATTCGAGTGACAA
		R	TGGGAGTAGACAAGGTACAACCC
Il6	mice	F	TAGTCCTTCCTACCCCAATTTCC
		R	TTGGTCCTTAGCCACTCCTTC
Il1b	mice	F	CCGTGGACCTTCCAGGATGA
		R	GGGAACGTCACACACCAGCA
MCP1	mice	F	TACAAGAGGATCACCAGCAGC

		R	ACCTTAGGGCAGATGCAGTT
β-actin	mice	F	GTGACGTTGACATCCGTAAAGA
		R	GCCGGACTCATCGTACTCC

Supplementary Table 2. Antibodies for immunoblot analyses.

**Antibody**	**Cat No.**	**Manufacturer**
GAPDH	60004-1-Ig	proteintech
MIF	Ab175189	Abcam
Bax	50599-2-lg	proteintech
Bcl2	3498	CST
C-Caspase3	9664	CST
p-ASK1	AP0058	Abclonal
ASK1	A3271	Abclonal
P-P38	4511	CST
P38	9212	CST
P-JNK	AP0631	Abclonal
JNK	66210-1-Ig	Proteintech

Supplementary Table 3. Primers for siRNA construction.

**Gene**	**Specie**		**Sequence5'---3'**
siMIF-1	human	F	5’-CGGACCAGCUCAUGACUUU-3’
		R	5’-AAAGUCAUGAGCUGGUCCG-3’
siMIF-2	human	F	5’- CCGCAACUACAGUAAGCUG -3’
		R	5’-CAGCUUACUGUAGUUGCGG-3’
siMIF-3	human	F	5’- GCCCGGACCGGGUCUACAU -3’
		R	5’-AUGUAGACCCGGUCCGGGC-3’
siNC	human	F	5’-UUCUCCGAACGUGUCACGUTT-3’
		R	5’-ACGUGACACGUUCGGAGAATT-3’
